# Advances in Subcellular Accumulation Design for Recombinant Protein Production in Tobacco

**DOI:** 10.34133/bdr.0047

**Published:** 2024-08-28

**Authors:** Shi-Jian Song, Hai-Ping Diao, Yong-Feng Guo, Inhwan Hwang

**Affiliations:** ^1^ Tobacco Research Institute, Chinese Academy of Agricultural Sciences, Qingdao, China.; ^2^ Beijing Life Science Academy (BLSA), Beijing, China.; ^3^ Department of Life Science, Pohang University of Science and Technology, Pohang, Republic of Korea.; ^4^ BioApplications Inc., Pohang, Republic of Korea.

## Abstract

Plants and their use as bioreactors for the generation of recombinant proteins have become one of the hottest topics in the field of Plant Biotechnology and Plant Synthetic Biology. Plant bioreactors offer superior engineering potential compared to other types, particularly in the realm of subcellular accumulation strategies for increasing production yield and quality. This review explores established and emerging strategies for subcellular accumulation of recombinant proteins in tobacco bioreactors, highlighting recent advancements in the field. Additionally, the review provides reference to the crucial initial step of selecting an optimal subcellular localization for the target protein, a design that substantially impacts production outcomes.

## Introduction

Plant molecular farming (PMF) has emerged as a promising approach in biotechnology. This technology harnesses the power of plants as bioreactors, transforming them into “green factories” for the production of valuable recombinant proteins such as therapeutical and industrial enzymes. PMF leverages the sophisticated biosynthetic machinery of plants to generate complex recombinant proteins, offering several advantages over established production methods like microbial fermentation and mammalian cell culture [1,2]. For instance, plants can be cultivated on a large scale at relatively low costs, reducing the overall expense of protein production. Plant-based systems can be easily scaled up by increasing the cultivation area, making them suitable for high-yield production. Plants do not harbor human pathogens, minimizing the risk of contamination with viruses, prions, or other harmful agents that can affect microbial and mammalian systems. Plants do not produce endotoxins, which are common in bacterial systems and can complicate purification processes and pose safety risks [3,4]. In addition to these commonly cited benefits, plants are able to provide significant flexibility and engineering potential, enabling tailored solutions for the production of diverse proteins to meet individualized customization needs.

A variety of plant species, including tobacco plant [5,6], carrot suspension cell [7–9], and rice seed [10–13], have been explored as platforms for producing and delivering commercialized recombinant proteins. Each type of these different plant bioreactor platforms offers distinct advantages in production efficiency, containment, scalability, and cost-effectiveness. For example, rice seeds possess specialized storage organelles that naturally facilitate protein accumulation, providing stability both within the plant and postharvest. Gt13a signal peptide (SP) was used to target recombinant human serum albumin into the protein storage vacuoles (PSVs) of endosperm cell, which resulted in the production yield of 2.75 g/kg brown rice [12]. The same strategy has been used for the production of Classical swine fever virus (CSFV) E2 dimer proteins [13] and Newcastle disease virus HN dimer proteins [10], which resulted in the production yield of 0.48 g/kg and 0.47 to 3.7 g/kg, respectively. The rice bioreactor, utilizing its unique PSV accumulation strategy in endosperm cells, can achieve significant yield of recombinant protein. However, it faces several limitations, including a lengthy cultivation cycle and the risk of contaminating food crops with genetically modified organisms.

Notably, tobacco species, particularly *Nicotiana tabacum* and *Nicotiana benthamiana*, which are nonfood and nonfeed crop status, produce large amounts of biomass in a relatively short life cycle, making them a prominent choice in PMF [14,15]. *N. benthamiana*, in particular, has a less robust RNA silencing pathway compared to other plants, reducing the degradation of foreign RNA. Additionally, its compromised basal immunity decreases the likelihood of an immune response to *Agrobacterium tumefaciens*, or other virus-based vectors used for gene delivery, enhancing the expression of introduced genetic material [16–18]. These immune deficiencies greatly enhance its suitability and popularity as a host for *Agrobacterium*-mediated transient expression of various recombinant proteins. This makes it particularly valuable for the rapid production of urgently needed pharmaceutical proteins, such as vaccines and antibodies, during a pandemic. Furthermore, *Agrobacterium*-mediated transient expression in tobacco is a powerful tool for quickly and efficiently producing recombinant proteins, allowing for the screening of efficient expression vectors, fusion tags, stabilization domains, and other regulatory elements within a few days.

Unlike rice seeds, which have a simple but robust endosperm cell PSV store strategy, tobacco cells require engineering for subcellular accumulation tailored to different recombinant protein properties. The unique intracellular organization influences the trafficking and posttranslational modification of recombinant proteins [19–21]. Various targeting signals, such as leader sequence for endoplasmic reticulum (ER) targeting, chloroplast transit peptides, and vacuolar targeting sequences, are used to direct proteins to desired destinations. Meticulous engineering of these signals has resulted in enhanced protein accumulation. Additionally, due to the difference in the ability to do N-glycosylation of recombinant proteins among organelles, the selection of appropriate subcellular compartments is crucial for production of recombinant proteins as pharmaceuticals.

While previous research has explored recombinant protein production in tobacco, a comprehensive review specifically focusing on subcellular targeting strategies is lacking. This review addresses this gap by summarizing strategies for directing recombinant proteins to 4 key compartments within tobacco cells: ER, vacuole, chloroplast, and apoplast. We exclude the cytoplasm due to its limitations for protein storage. The cytoplasm is an open and dynamic environment, characterized by numerous competing cellular processes, and hinders efficient protein accumulation. Additionally, the complex protein degradation machinery in the cytoplasm poses a significant risk of unintended breakdown of the desired protein, leading to lower yields. By examining these targeted approaches, this review sheds light on the initial steps for selecting the optimal location for specific recombinant protein production in tobacco plants.

## Recombinant Proteins Accumulated in ER

The ER in plants is a dynamic organelle crucial for various cellular functions. It serves as a hub for interorganelle communication, playing a vital role in the exchange of proteins, ions, and metabolites between different organelles [22–25]. Understanding the ER’s functions not only advances basic cell biology but also holds significance for biotechnological applications in recombinant protein production.

Accumulating recombinant proteins in the plant ER can be achieved by incorporating an N-terminal ER targeting or secretion SP together with a C-terminal retention sequence [19,26]. This involves mechanisms and benefits that have been well studied. First, directing the synthesis of foreign proteins to the ER, rather than the cytosol, minimizes proteolytic breakdown [27]. Second, the ER contains abundant molecular chaperones to aid the folding of proteins [28,29]. Third, the ER accumulation of proteins prevents the modification of N-glycan at the Golgi apparatus, thereby N-glycosylated proteins having homogeneous high-mannose glycan forms [30–33].

The ER has 2 main pathways for folding protein substrates. The first pathway is the general folding pathway, primarily facilitated by BiP (the ER homolog of the 70-kDa heat shock proteins, Hsp70) and P4HB (the founding member of the protein disulfide isomerase family). The second pathway is specific for glycoproteins and is mediated by the lectin chaperone calreticulin (CRT) and its membrane-bound homolog calnexin, which bind to monoglucosylated N-glycans on substrate proteins to aid their folding [34]. In *N. benthamiana*, heat shock treatment by placing the infiltrated plants in a 37 °C incubator for 30 min at 1 d postinfiltration significantly increased the expression of ER-accumulated S_ct_ (the trimeric ectodomain of the severe acute respiratory syndrome coronavirus 2 [SARS-CoV-2] spike protein). This suggests that the heat shock induces endogenous chaperone machinery, which aids the accumulation of recombinant proteins in the ER. Additionally, coexpressing human CRT in the ER increased the overall yield of S_ct_ by up to 3.51-fold. However, simultaneous application of heat shock-induced chaperone machinery and human CRT coexpression did not further improve S_ct_ accumulation, indicating that relying solely on protein-assisted folding to increase accumulation has an upper limit. Moreover, human CRT did not enhance the expression of S_ct_-3P, a well-folded version of S_ct_, suggesting that chaperones may not assist well-folded proteins [35].

N-glycosylation occurs in the ER of both plants and mammals. The structure of N-glycans on nascent proteins are identical in the ER. When these proteins arrive to the Golgi apparatus, the N-glycans are subjected to species-specific modifications [26]. In plants, this involves the addition of β(1,2)-linked xylose and core α(1,3)-linked fucose residues, while in mammals, it includes the addition of β (1,4)-linked galactose and sialic acid residues [36]. Therefore, accumulation of recombinant proteins to the ER using an ER retention motif is commonly used to prevent the incorporation of plant-specific sugar residues. Additionally, the formation of glycan structures with terminal mannose residues in the ER enables recombinant proteins to target mannose-specific surface receptors on macrophages, which is believed to enhance antigen uptake for recombinant vaccines by macrophages [37].

Until now, most recombinant proteins accumulating in plant ER have relied on the addition of an ER retention/retrieval sequence, like HDEL or KDEL, at their C-terminus. This strategy has successfully increased the accumulation of various proteins, including interleukin-4 and interleukin-6 [38,39], hemagglutinin H5 and H9 [40], CSFV E2 [41], SARS-CoV-2 Spike [35], carbonic anhydrases [42], and monoclonal antibody (mAb) CO17-1A [43] in tobacco cells (Table 1). ER retention/retrieval sequence receptors at the Golgi complex perform this recognition to retrieve C-terminal tagged proteins into the ER lumen through a process mediated by coat protein complex I (COPI). However, it is possible that the machinery gets submerged by saturation of HDEL/KDEL-containing proteins. If these receptors are saturated or processed, tagged recombinant proteins might either be secreted or transported to lytic vacuoles (LVs). For example, the anti-HIV antibody 2F5 heavy chain, fused with the C-terminal retention signal KDEL (2F5-HDEL), undergoes processing that removes the retention signal, resulting in the secretion of the antibody into the intercellular fluid [44]. Additionally, sporamin, a well-characterized storage protein naturally found in sweet potato vacuoles, was detected in the vacuole of BY2 cells rather than ER even when fused with the ER-retention signal HDEL (SpoHDEL), suggesting that SpoHDEL escapes the ER retention machinery [45]. Compared to HDEL/KDEL fused recombinant proteins that successfully target ER, there are few examples of ER off-target, but it does exist. In our past studies, combining a classical N-terminal ER-targeting SP with a C-terminal HDEL/KDEL sequence to recombinant proteins (e.g., AtBIP1 + HDEL/KDEL) has proven highly effective in achieving accurate ER targeting and retention. Notably, the first improved plant-based vaccine for CSFV (HERBAVAC CSF Green Marker) is based on the ER accumulation via AtBIP1 leading and HDEL retention [46].

**Table 1.** List of recombinant proteins accumulated in the tobacco ER

Protein	SP	Expression level	Tobacco species	References
IL-4	Barley α-amylase	0.1% TSP	*N. tabacum*	[38]
IL-6	*AtBIP1*	18.49 mg/kg FW	*N. benthamiana*	[39]
H5/H9 trimer	*AtBIP1*	150–250 mg/kg FW	*N. benthamiana*	[40]
CSFV E2	*AtBIP1*	302 mg/kg FW	*N. benthamiana*	[46]
SARS-CoV-2 spike trimer	*AtBIP1*	106 mg/kg FW	*N. benthamiana*	[35]
Carbonic anhydrases	*AtBIP1*	350 mg/kg FW	*N. benthamiana*	[42]
mAb CO17-1A	Zera	340 mg/kg FW	*N. tabacum*	[43]
F1-V	Zera	230±20 μg/g TSP	*N. benthamiana*	[47]
		8.5±0.2 mg/g TSP	NT1 cells	

IL-4, interleukin-4; IL-6, interleukin-6; H5/H9, hemagglutinin 5 and 9; F1-V, *Yersinia pestis* F1 antigen; FW, fresh weight; TSP, total soluble protein

Beyond the standard C-terminal HDEL/KDEL retention signal, the formation of protein bodies (PBs) within the ER can significantly enhance recombinant protein accumulation. This process is often triggered by specific protein tags, such as the N-terminal proline-rich domain of γ-zein (Zera). The Zara-associated PB accumulation results in around 5-fold increase of recombinant *Yersinia pestis* F1-V antigen in *N. benthamiana* leaves upon *Agrobacterium*-mediated transient expression [47]. Once PBs arise for Zera-ECFP (enhanced cyan fluorescent protein), they grow in size over time, reaching their maximum size in *N. benthamiana* leaves at 10 d postinfiltration [48] (Table 1).

## Recombinant Proteins Accumulated in the Vacuole

The plant vacuole stands as a multifunctional and dynamic organelle crucial for upholding turgor pressure, storing and amassing inorganic ions, amino acids, proteins, sugars, and secondary compounds, maintaining cell homeostasis, facilitating signal transduction, and fostering plant development. In mature cells of tobacco leaves, the plant vacuole dominates, occupying a substantial 80% to 90% of the cell volume [49]. This substantial space presents tremendous potential for the storage of recombinant proteins.

There are 2 types of vacuoles that are identified as having separate and specific functions: PSVs and LVs [50]. PSVs present in seed and some tissues can store large amounts of foreign proteins for long periods without degradation, while in contrast, LVs are found in most of cells in vegetative tissue where proteins are subjected to degradation like lysosomes in animal cells. Even so, several foreign proteins including avidin [51], endolysin [52], human collagen [53], α-galactosidase A [54], human complement factor C5a [55], interleukin-6 [56], and mAb 14D9 [57] (Table 2), can accumulate in the vacuole of tobacco leaves or tobacco suspension culture cells, suggesting that the central LV in leaf tissues can be a suitable destination for certain proteins.

**Table 2.** List of recombinant proteins accumulated in tobacco vacuole

Protein	Signal origin	Expression level	Tobacco species	References
Avidin	PPI-I	1.5% TSP	*N. benthamiana*	[51]
Endolysin	PPI-I	160 mg/kg FW	*N. benthamiana*	[52]
Human collagen	BTPA	2% TSP	*Tobacco*	[53]
α-Galactosidase A	TCA	302 mg/kg FW	BY-2 cells	[54]
C5a	AFVY	10.62 mg/kg FW	*N. tabacum*	[55]
IL-6	AFVY	5.21 μg/kg TSP	*N. tabacum*	[56]
mAb 14D9	A11G	1.57%–1.73% TSP	*N. tabacum*	[57]

PPI-I, potato proteinase inhibitor I; BTPA, barley thiol protease aleurain; TCA, tobacco chitinase A; C5a, human complement factor 5a. AFVY, the C-terminal tetrapeptide of phaseolin; A11G, amaranth 11S globulin (KISIA Ct and NIFRGF ss)

Different vacuolar sorting signals (VSSs) have been described for vacuolar targeting in tobacco. For instance, the sequence-specific (ss) signals consist of short, defined amino acid motifs, such as NPIR or NPIXL [57,58]. Notably, the position of these motifs within the protein sequence does not affect their function. By contrast, hydrophobic C-terminal signals (Ct) lack a specific consensus motif but are invariably located at the C-terminus of the protein, suggesting that location is critical for vacuolar targeting [59]. Fusing the heavy chain of mAb 14D9 with either Ct VSS (KISIA) or ss VSS (NIFRGF) resulted in 10- to 15-fold higher vacuolar accumulation of mAb compared to the secreted mAb version in *N. benthamiana*, while Ct VSS (KISIA) and ss VSS (NIFRGF) themselves did not induce differences in production yield [57], suggesting that the central vacuole can serve as an appropriate compartment for the efficient production of antibodies.

Proteins destined for vacuoles enter the plant secretory pathway via an SP and can take different trafficking routes. The conventional transport pathway that is the most characterized involves initial export from the ER via COPII vesicles [60,61]. Following this, the proteins transit through the Golgi apparatus where the N-glycans of proteins are modified to the complex type. At the trans Golgi network, the vacuolar proteins are sorted and undergo post-Golgi transport to reach the vacuole. An alternative trafficking route that is unconventional but occurred entirely independent of COPII machinery, has also been identified. Proteins are transported directly from the ER to the vacuole and bypass the Golgi apparatus [62]. For example, a mouse immunoglobulin G1 (IgG1) fused to a ss VSS and Ct-VSS of amaranth 11S globulin produced in *N. benthamiana* is decorated with 75% Man7 and Man8 glycans supporting a direct transport bypassing the Golgi [57], while a mouse IgG1 fused with the VSS of sporamin produced in suspension-cultured tobacco BY2 cells is predominantly decorated with Man3XylFucGlcNAc2 glycans [63], supporting a conventional transport passing Golgi.

Taliglucerase alfa (Elelyso) is the first Food and Drug Association-approved plant-based recombinant protein for human use. This recombinant human glucocerebrosidase utilizes a C-terminal VSS (DLLVDTM) derived from tobacco chitinase A to achieve vacuolar targeting in carrot suspension cells. In this instance, vacuolar accumulation also facilitates the presence of Man3XylFucGlcNAc2 glycan [7]. Notably, the recombinant human glucocerebrosidase produced in the vacuole of carrot cells naturally contains terminal mannose residues on its complex glycans. Unlike Cerezyme, it does not require the in vitro exposure of mannose residues while still displaying comparable enzymatic activity and uptake into macrophages.

## Recombinant Proteins Accumulated in Chloroplasts

Plant chloroplasts originated from a symbiotic relationship between a eukaryotic ancestor and cyanobacteria, retaining their own genetic material and gene expression machinery [64]. They are essential organelles responsible for the biosynthesis of starch, lipids, amino acids, nucleotides, and other metabolites. Chloroplasts are naturally equipped to handle the highest level of native proteins related to photosynthesis in tobacco leaf tissues. This capacity potentially can be harnessed to accumulate large quantities of recombinant proteins. Chloroplasts are highly abundant in mesophyll cells, occupy a large volume, and are an attractive organelle for storing plant-derived recombinant proteins.

There are 2 primary strategies for accumulating recombinant proteins in chloroplasts. The first involves directly inserting the desired gene into the chloroplast genome using homologous recombination. This gene is then transcribed and translated within the chloroplasts. The second strategy relies on nuclear transformation, where the gene is integrated into the plant nuclear genome. The resulting protein is subsequently transported from the cytoplasm into the chloroplasts. For simplicity, we refer to these approaches as “chloroplast transformation” and “nuclear transformation”, respectively.

In case of chloroplast transformation, chloroplasts lack gene silencing mechanisms, resulting in more stable and consistent expression of foreign genes. In addition, the chloroplast environment provides suitable conditions for protein folding and disulfide bond formation, crucial for proper protein function [65]. Furthermore, chloroplast-derived transgenes exhibit minimal risk of transfer to the environment due to their low transmission rates through pollen. Notably, chloroplast transformation has achieved a maximum recombinant β-glucosidase production level of 75% of total soluble protein (TSP) [66]. Generally, conventional methods typically yield up to approximately foreign protein at 5% to 20% TSP for certain cases [67]. Targeted integration of foreign genes into the chloroplast genome has been successfully achieved via biolistic bombardment [68,69]. A significant portion of recombinant protein production achieved through tobacco chloroplast transformation relies on this method such as the endoglucanase [67,69], superoxide dismutase [70], epidermal growth factor [71,72], cholera toxin B subunit [73], and human papillomavirus L1 virus-like particle (VLP) [74] (Table 3). While chloroplast transformation is effective for producing certain recombinant proteins, it has limitations compared to nuclear transformation. Generating stable, high-yielding transgenic plants through chloroplast transformation is often time-consuming due to technical challenges. Furthermore, chloroplasts lack the cellular machinery necessary for protein glycosylation that is required for some pharmaceutical proteins.

**Table 3.** List of recombinant proteins accumulated in tobacco chloroplast

Protein	Expression pattern	Transit peptide	Expression level	Tobacco species	References
β-Glucosidase	Chloroplast		75% TSP	*N. tabacum*	[66]
TetC-Cel6A	Chloroplast		28% in green leaves	*N. tabacum*	[67]
SOD	Chloroplast		9% TSP	*N. tabacum*	[70]
EGF	Chloroplast		1.57±0.05 g/kg FW	*N. tabacum*	[72]
CTB	Chloroplast		7.49 mg/g FW	*N. tabacum*	[73]
HPV L1 VLP	Chloroplast		24% TSP	*N. tabacum*	[74]
HIV-1 p24	Nucleus	rbcS1-TP	1 mg/kg FW	*N. benthamiana*	[76]
HPV-16 L1 VLP	Nucleus	rbcS1-TP	6–8 mg/kg FW	*N. benthamiana*	[79]
HPV-16 L1 VLP	Nucleus	rbcS1-TP	11% TSP	*N. benthamiana*	[77]
HPV-16 L1/L2 chimera	Nucleus	rbcS1-TP	1.2 g/kg FW	*N. tabacum*	[78]

TetC-Cel6A, endoglucanase Cel6A; EGF, epidermal growth factor; CTB, cholera toxin B subunit; SOD, superoxide dismutase; HPV L1 VLP, human papillomavirus L1 virus like particle; HIV-1 p24, human immunodeficiency virus type I p24 capsid subunit; HPV-16 L1/L2 chimera, human papillomavirus type 16 L1/L2 chimeras; rbcS1-TP, transit peptide from *rbcS1*

In case of nuclear transformation, this method utilizes a transgene encoding the recombinant protein fused to a chloroplast transit peptide. The transgene can be either transiently expressed in the nucleus or stably integrated into the plant nuclear genome. The translated protein, harboring the N-terminal chloroplast transit peptide, is then imported into the chloroplasts from the cytosol. This import process is mediated by translocon complexes located at the outer and inner envelope membranes of chloroplast, termed TOC and TIC, respectively [75]. Transient expression in the nucleus followed by protein targeting to the chloroplasts offers a significant time advantage for recombinant protein production compared to the chloroplast transformation, which has gained traction, as evidenced by several studies employing it, such as HIV-1 p24 [76] and HPV-16 VLP [77–79] (Table 3). However, it is important to note that since these recombinant proteins are synthesized in the cytosol, their final accumulation within chloroplasts will depend on both the chloroplast protein import capacity and ubiquitin-dependent turnover processes [80]. Thus, this eukaryotic posttranslational modification system typically yields lower recombinant protein levels compared to chloroplast transformation, with expression generally falling below 10% TSP [81]. For example, HPV L1 VLP expressed in chloroplasts reach approximately 24% TSP [74], whereas expression via the nucleus–chloroplast targeting approach typically yields only around 11% TSP [77].

## Recombinant Proteins Accumulated in Apoplast

The plant apoplast is a crucial space between the cell membrane and the cell wall that plays a significant role in various biological processes. It serves as the primary site for pathogen recognition, triggering immune responses involving the secretion of molecules like proteases, proteins related to immunity, small RNAs, and secondary metabolites [82–84]. Additionally, the apoplasts are considered to be a good place for recombinant protein accumulation in view of the large volume of extracellular space without intracellular proteolytic organelle-mediated protein degradation [85].

Even the analysis of the plant secretome has revealed that more than 50% of the endogenous secreted proteins lack an SP [86]; a well-studied SP rather than the native SP is thought to be necessary to direct recombinant proteins to this specific extracellular location. For example, the signal sequence and the first 2 amino acids derived from the tobacco PR1b protein, when fused to the bacterial protein ChiA, significantly enhanced its secretion efficiency compared to ChiA with its native signal sequence when expressed in plant cells [87]. The production of HRP C1a using GE derived from *N. tabacum* β-D-glucan exohydrolase shows a better secretion rate in BY2-cell system than its native SP and another one derived from peroxidase [88]. IgG is a kind of native secretion protein in animal cells but not always adaptive for the secretion in plant cells. While some studies have replaced the signal sequences for better secretion [89], most have retained the native SP, leading to detectable IgG in the plant apoplast, PVC, and ER [90]. Three potential explanations for these observations arise: (a) A part of the recombinant IgG in folding intermediates or in misfolded form is bonded by the chaperones BiP in the ER [91]. (b) The ER–Golgi–apoplast pathway is a classic secretion pathway. It makes sense that recombinant IgGs could be detected along the entire pathway unless the protein synthesis is blocked for a while. (c) The movement of proteins from the ER to Golgi and their subsequent sorting within the Golgi is meticulously controlled by vesicles and associated proteins like COP and adaptor protein complexes. Inefficiencies in this process could hinder IgG progression.

For tobacco suspension cells, protein secretion offers advantages due to simplified purification methods via medium collection. Proteins directed to the secretory pathway are typically released into the culture medium after traversing the cell wall. However, traversing efficiency varies based on protein size and physicochemical properties. Smaller proteins (<30 kDa) are generally completely secreted [92,93], while larger ones were considered retained to varying degrees. Notably, even large proteins like antibodies can be efficiently secreted and pass the cell wall [89], while some small ones like green fluorescent protein (GFP) fused major ampullate silk proteins (MaSp) remain trapped [94], suggesting that factors beyond size, such as charge and hydrophobicity, also play a role. BY-2 cell-produced and apoplast-secreted platforms have emerged as a versatile platform for the successful expression of a broad range of functional proteins, including interferon a2b [95], human a-L-iduronidase [96], anti-HIV-antibody 2G12 [97], human growth hormone [98], anti-vitronectin antibody M12 [99], human α1-antitrypsin [100], ORF8 of SARS-CoV-2 [101], and stem cell factor [102] (Table 4).

**Table 4.** List of recombinant proteins accumulated in tobacco apoplast

Protein	Signal origin	Expression level	Tobacco species	References
Interferon α2b	Extensin	28 mg/l	BY-2 cells	[95]
hIDUA	Proaleurain	10 mg/l	BY-2 cells	[96]
2G12	Native SP	8 mg/l	BY-2 cells	[97]
rhGH-(SO)10	Extensin	16–35 mg/l	BY-2 cells	[98]
rhGH-(SO)10-EGF		15–32 mg/l		
M12	mAb24	20 mg/l	BY-2 cells	[89]
Human α1-antitrypsin	Extensin	34.7±4.3 mg/l	BY-2 cells	[100]
ORF-8	Native SP	8.8±1.4 mg/l	BY-2 cells	[101]
(SP)20-SCF	Extensin	2.5 mg/l	BY-2 cells	[102]
SCF-(SP)20		1.4 mg/l		
H5 VLP	Native SP	50 mg/kg FW	*N. benthamiana*	[5]
SARS-CoV-2 VLP	Native SP	24–28 mg/kg FW	*N. benthamiana*	[105]
SARS-CoV-2 VLP	Plant	N/A	*N. benthamiana*	[107]

hIDUA, human lysosomal enzyme a-iduronidase; 2G12, human anti-HIV antibody; rhGH-(SO)10, rhGH was expressed with 10 repeats of the AGP glycomodule Ser-Hyp (SO) at the C-terminus; rhGH-(SO)10-EGF, rhGH-(SO)10 as an AGP-enhanced green fluorescent protein fusion; M12, anti-vitronectin antibody; ORF-8, SARS-CoV-2 open reading frame 8; (SP)20-SCF, 20 tandem repeats of the “Ser-Pro” motif fused to the N-terminus of stem cell factor gene; SCF-(SP)20, 20 tandem repeats of the “Ser-Pro” motif fused to the C-terminus of stem cell factor gene

In whole tobacco plants, the recombinant proteins that primarily secreted to the apoplast and accumulate in the extracellular space will undergo endocytosis to a limited extent, but it does not significantly affect the overall accumulation [103]. The secreted soluble proteins can be efficiently extracted from the apoplast fluid of tobacco leaves using a simple low-speed centrifugation of intact leaves [104]. This method is advantageous to the downstream processing due to less volume of extracts and the rare release of intracellular proteins. However, the large protein complexes, such as the influenza VLP produced by transient expression in *N. benthamiana*, require conventional extraction procedures. It is clearly observed by electronic microscopy that they are accumulated between the plasma membrane and the cell wall [5]. The apoplast targeting in tobacco plants has emerged as a promising platform for the production of VLP vaccines via vesicles budding, demonstrating its potential for industrial applications [105,106] (Table 4). Notably, the COVID-19 (coronavirus disease 2019) VLP vaccine made by Medicago (Covifenz), is the first approved plant-based human vaccine [107]. Glycoproteomic analysis of secreted SARS-CoV-2 VLPs produced in *N. benthamiana* revealed occupancy of 20 out of the predicted 22 N-glycosylation sites with complex plant N-glycans, while 1 site contained oligomannoses [108]. However, the absence of nonmammalian epitopes, such as core β (1,2)-xylose and α (1,3)-fucose residues of SARS-CoV-2 VLP vaccine, associated with plant N-glycosylation have not developed any allergic or hypersensitivity reactions in subjects in the Phase 1 randomized trial [107].

Recombinant proteins targeted to the plant apoplast do face challenges, particularly related to protease processing that can compromise their structural integrity to certain recombinant proteins [109]. For example, the recent study suggested that SBT5.2s are the major active extracellular subtilases processing IgG antibody 2F5 in the *N. benthamiana* apoplast [110]. Moreover, the recombinant SARS-CoV-2 VLPs accumulated in the apoplast of *N. benthamiana* leaves were processed to be incomplete [105]. To these challenges, coexpression of protease inhibitors has emerged as a promising strategy to stabilize proteins in the leaf apoplast, potentially leading to enhanced protein accumulation and integrity [111].

## Which to Choose?

Here, we summarized the pros and cons of different subcellular accumulation (Figure C). The ER provides a conducive environment for proper protein folding and assembly, with the presence of chaperones and folding enzymes that assist in forming disulfide bonds and achieving the correct tertiary structure. It can perform homogeneous glycosylation, essential for the functionality and stability of many therapeutic proteins. However, overloading the ER can lead to ER stress and reduced yields, necessitating careful management of the expression levels.

Vacuoles can pose challenges for recombinant protein stability due to their protease-rich environment, which can degrade proteins. However, some proteins are naturally stable in vacuoles, indicating that specific proteins may be suitable for this compartment. Stabilization strategies are essential when targeting proteins to vacuoles to prevent degradation and maintain functional protein levels.

Chloroplasts offer a high-yield environment for recombinant protein production due to their capacity for high rates of transcription. The plastid genome can accommodate multiple copies of the gene of interest, further boosting protein yield. However, the chloroplast transformation is time-consuming due to technical challenges, while the nuclear-expressed and transit peptide-mediated translocation is less efficient.

The apoplast is an extracellular environment that supports the formation of membrane associated VLP. Proteins secreted into the apoplast can be easily harvested from the extracellular fluid, simplifying the purification process and potentially increasing yield. However, the presence of proteases in the apoplast can process recombinant proteins, requiring the use of protease inhibitors or engineering protease-resistant protein variants to maintain protein integrity and yield.

Optimizing subcellular localization for individual target proteins, rather than employing a universal strategy, is vital at the beginning of target protein design. Several studies have directly investigated the impact of subcellular targeting on recombinant protein expression level. For instance, comparisons have been made between the accumulation of GFP and GB1-GFP when transiently expressed in *N. benthamiana* [112]. This study revealed that accumulation in the ER resulted in slightly higher fluorescence intensity compared to chloroplast accumulation, with both significantly exceeding stronger than in cytosol. Similarly, research has demonstrated that stable expression of interleukin-6 in *N. benthamiana* yielded significantly higher protein levels when targeted to the ER compared to the apoplast or vacuole [56]. Roughly, the ER and chloroplasts are often preferred for their conducive environments for protein folding and high expression levels, respectively. However, the specific requirements of the target protein, such as necessary posttranslational modifications and resistance to degradation, must be carefully considered to ensure successful production.

Here, we provide insight for the subcellular targeting strategies of different types of proteins: (a) The large and complex glycoproteins that require chaperone-assisted folding and mammalian-like N-glycosylation patterns may give priority to ER retention. (b) Proteins tolerant of acidic environments, particularly those naturally localized to human lysosomes, can benefit from vacuolar accumulation. (c) Proteins that do not require extensive posttranslational modifications and readily fold correctly may be a good target for expression via chloroplast transformation. Notably, chloroplasts can also facilitate the assembly of coat protein to VLPs. (d) The extracellular space is an alternative option for proteins requiring membrane-mediated budding, such as certain VLPs. Within this review article, we present illustrative figures depicting representative proteins and their predominant glycosylation patterns associated with specific subcellular compartments for your reference (Figure).

**Figure.**
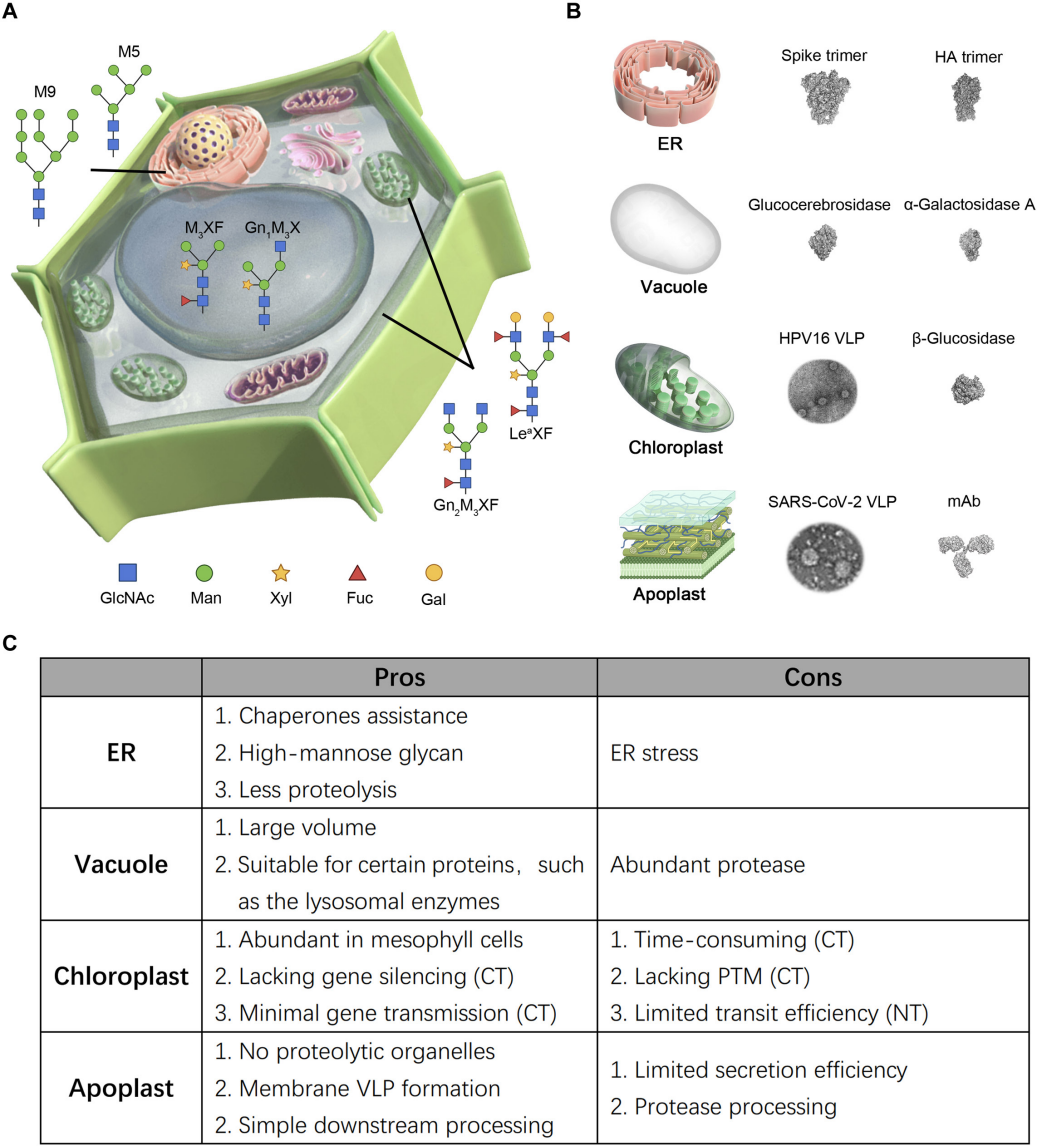
Representative proteins and their predominant glycosylation patterns associated with specific subcellular compartments, along with the pros and cons. (A) Representative predominant glycosylation patterns in the specific subcellular compartments. GlcNAc (Gn), N-acetylglucosamine; Man (M), mannose; Xyl (X), xylose; Fuc (F), fucose; Gal, galactose, Le^a^, Lewis a. (B) Representative proteins in specific subcellular compartments. The Cryo-EM data of representative VLPs and the PDB structure of representative proteins are flanked with the 4 subcellular compartments. (C) The pros and cons of different subcellular accumulation in tobacco. PTM, posttranslation modification; CT, chloroplast transformation; NT, nuclear transformation.

## Challenges and Future Perspectives

While PMF offers significant promise for industrial production, several key challenges must be addressed to fully realize its potential: (a) Adaptable production levels. Achieving consistent and scalable production levels for diverse recombinant proteins remains a crucial hurdle. (b) Comparable quality. Ensuring that the recombinant proteins possess comparable quality attributes (e.g., posttranslational modifications) to their authentic counterparts is essential. (c) Effective downstream processing costs. Minimizing downstream processing costs associated with protein purification and isolation is critical for economic feasibility. To address the 3 major scientific and technical challenges in the PMF field, future studies should focus on expression design, plant remodeling, and protein purification.

Expression design has been the most extensively studied during the past few decades, encompassing both gene-level and protein-level strategies. At the gene level, foreign genes should be optimized to match the tobacco codon preferences, reducing sequence complexity and minimizing secondary structures for greater genetic stability, which is crucial for long-term experiments or commercial production [113]. Robust 5′- and 3′-untranslated regions are necessary to stabilize mRNA and enhance translation levels [114], while suppressors are needed to block RNA silencing, enhancing the stability and accumulation of transgene mRNA in transient expression systems [115]. At the protein level, designing appropriate subcellular compartment targeting and storage SPs ensures better protein storage and precise posttranslational modification. Adding solution-promoting tags, high-glycosylation domains, and stabilization domains helps recombinant proteins fold correctly, improving expression levels. Additionally, coexpression of molecular chaperone proteins assists in the folding of recombinant proteins and increases their expression.

There has been less research on plant chassis remodeling compared to expression design. However, recent years have seen the creation of several transgenic tobacco lines to address plant-specific glycosylation modifications. CRISPR-Cas9-engineered *N. benthamiana* [116] and BY-2 cells [117], for example, have been developed to completely lack α1,3-fucose and β1,2-xylose residues. These engineered plants and plant cells are generally well suited for producing pharmaceutical proteins that require proper glycosylation. Furthermore, according from various challenges we have faced in the expression and purification of recombinant proteins from tobacco, we believe that remodel diverse tobacco plant chassis is crucial for the future development of this field. For example, (a) modifying plant reactors to have low-efficiency protease processing to reduce the proportion of recombinant protein processed by plant endogenous proteases. (b) Establishing plant reactors that can efficiently allocate host synthetic resources to increase recombinant protein yield. (c) Creating plant reactors without polyphenol contamination to minimize polyphenol pollution during the purification process.

In the field of PMF, the eventual commercialization of biomanufacturing is a key indicator of progress. Currently, the number of recombinant protein drugs available from plant-based systems is significantly lower compared to those from nonplant systems. The closure of Medicago in 2023, resulting in the withdrawal of SARS-CoV-2 VLP vaccine from the market, has negatively impacted the field, although this was unrelated to technological issues [118]. All these factors remind us that beyond scientific and technical considerations, public opinion on tobacco products and stringent government regulations on using transgenic plants to produce recombinant proteins play a crucial role in industrialization. Adhering to rigorous regulatory frameworks, engaging with the public through transparent communication, and establishing robust safety protocols for handling and disposal are essential to overcoming public and governmental concerns.
